# Supplementary material for: Factors influencing participant compliance in acupuncture trials: An in-depth interview study
Source: PLoS One. 2020 Apr 16;15(4):e0231780. doi: 10.1371/journal.pone.0231780 (PMC7162473; doi:10.1371/journal.pone.0231780)
Supplement: S1 Table — (DOCX) [file pone.0231780.s001.docx]

**Supporting Table 1 Consolidated criteria for reporting qualitative research (COREQ): a 32-item checklist for interviews and focus groups**

| **No. Item** | **Guide questions/description** |
| --- | --- |
| **Domain 1: Research team and reflexivity**  **Personal Characteristics** | |
| 1. Interviewer / facilitator | Which author(s) conducted the interview of focus group?  The interviewers were two postgraduate students majoring in Evidence-based Medicine and trained in qualitative research (XLL and YJZ, females). Page 3 |
| 1. Credentials | What were the researcher’s credential? E.g. PhD. MD  Postgraduate students (BSc.) majoring in Evidence-based Medicine Page 3 |
| 1. Occupation | What was their occupation at the time of the study?  Postgraduate students majoring in Evidence-based Medicine Page 3 |
| 1. Gender | Was the researcher male or female?  XLL and YJZ were both females Page 3 |
| 1. Experience and training | What experience or training did the researcher have?  They were trained in how to conduct interviews by other research staff experienced in qualitative research (XL). Page 3 |
| **Relationship with participants** | |
| 1. Relationship established | Was a relationship established prior to study commencement?  They had no connection with the participants prior to the study. Page 3 |
| 1. Participant knowledge of the interviewer | What did the participants know about the researcher? E.g. personal goal, reasons for doing the research  The interviewees were informed about the researchers’ background, occupation and credentials. They were described the purpose of the research, signed the informed consent, and could withdraw from the interviews at any time during the research process. Page 2 |
| 1. Interviewer characteristics | What characteristics were reported about the interviewer/facilitator? E.g. Bias, assumptions, reasons and interests in the research topic  The interviewers are postgraduate students major in Evidence-based Medicine, female. They were only involved in conducting the interviews, and were not acupuncturists. Page 2 |
| **Domain 2: study design**  **Theoretical framework** | |
| 1. Methodological orientation and theory | What methodological orientation was stated to underpin the study? E.g. grounded theory, discourse analysis, ethnography, phenomenology, content analysis  Thematic content analysis Page 3 |
| **Participant selection** | |
| 1. Sampling | How were participants selected? E.g. purposive, convenience, consecutive, snowball  The sampling methods of the survey were convenience sampling and continuous sampling. Page 2 |
| 1. Method of approach | How were participants approached? E.g. face-to-face, telephone, mail, email  By telephone. Page 3 |
| 1. Sample size | How many participants were in the study?  There were 10 participants. The sample size was determined according to the principle of information saturation, with recruitment stopped when no new themes emerged. Page 2 |
| 1. Non-participation | How many people refused to participate or dropped out? Reasons?  40 people were contacted, of which 30 people declined to participate in this study because they had no time or interest. Page 3 |
| **Setting** | |
| 1. Setting of data collection | Where was the data collected? E.g. home, clinic, workplace  Most interviews were conducted in a separate doctor's office, with the exception of one interviewee who used video interview for their convenience. Page 2 |
| 1. Presence of non-participants | Was anyone else present besides the participants and researchers?  No; each interviewee was interviewed individually. Page 2 |
| 1. Description of sample | What are the important characteristics of the sample? E.g. demographic data, date  The 10 participants were aged between 27 to 65 years old. Two males and eight females; seven local (Beijing) and three non-local. Four participants were employed, three were unemployed and three were retired. Two patients had to pay for their own treatment, and the other eight people had medical insurance. Respondents were recruited from three acupuncture clinical trials in two hospitals, and assigned numbers according to the organization and the order of selection. The basic characteristics of the ten interviewees are shown in Table 1. Page 3 |
| **Data collection** | |
| 1. Interview guide | Were questions, prompts, guides provided by the authors? Was it pilot tested?  We developed the outline of the interview (see Appendix 1) based on the literature, the results of the survey and the pre-interview. Page 2 |
| 1. Repeat interviews | Were repeat interviews carried out? If yes, how many?  There was no participant withdrawal after consent, and no repeat interviews were carried out. Page 4 |
| 1. Audio/visual recording | Did the research use audio or visual recording to collect the data?  Two researchers (XLL and YJZ) collected audio data, and transcribed the recordings into a transcript. Page 3 |
| 1. Field notes | Were field notes made during and/or after the interview or focus group?  No. |
| 1. Duration | What was the duration of the interviews or focus group?  Each interviewee was interviewed individually for about 30 minutes, on average. Page 2 |
| 1. Data saturation | Was data saturation discussed?  The analysis of the 9th interview produced no new codes or concepts. We concluded that saturation had been reached. Page 4 |
| 1. Transcripts returned | Were transcripts returned to participants for comment and/or correction?  Transcripts were not returned to the participants. |
| **Domain 3: Analysis and findings**  **Data analysis** | |
| 1. Number of data coders | How many data coders coded the data?  After transcription, two researchers (XLL and HJC) independently coded the transcription data, discussed and merged codes after each interview, and consulted another researcher (JPL) if they could not reach a consensus. Page 3 |
| 1. Description of the coding tree | Did authors provide a description of the coding tree?  Table 2 presents the thematic framework of the themes. |
| 1. Derivation of themes | Were themes identified in advance or derived from the data?  Themes were derived from the interview data. Page 3 |
| 1. Software | What software, if applicable, was used to manage the data?  No software was used. |
| 1. Participant checking | Did participants provide feedback on the findings?  Participant feedback was not undertaken. |
| **Reporting** | |
| 1. Quotations presented | Were participant quotations presented to illustrate the themes / findings? Was each quotation identified? E.g. participant number  Participant quotations were used to illustrate themes, with participants given numbers, according to their organization and order of selection, to anonymize their identity. Page 3 |
| 1. Data and findings consistent | Was there consistency between the data presented and the findings?  We believe there is consistency between the presented data and the findings. |
| 1. Clarity of major themes | Were major themes clearly presented in the findings?  Yes, see Results section>Overview. Page 3 |
| 1. Clarity of minor themes | Is there a description of diverse cases or discussion of minor themes?  Within the major themes we have also discussed separate sub-themes. See Page 3-7 and in Table 2. |
