# Supplementary material for: Factors influencing participant compliance in acupuncture trials: An in-depth interview study
Source: PLoS One. 2020 Apr 16;15(4):e0231780. doi: 10.1371/journal.pone.0231780 (PMC7162473; doi:10.1371/journal.pone.0231780)
Supplement: S2 Table — (DOCX) [file pone.0231780.s002.docx]

**Supporting table 2 Interview outline**

| 1. **Subject one: Knowledge of clinical trials** | |
| --- | --- |
| 1.1 | Have you ever known about clinical trials before? How did you learn about clinical trials for acupuncture? |
| 1.2 | Have you participated in any other clinical trials before? How do you feel about the clinical trials you participated in? |
| 1.3 | How did you come into contact with clinical trials? What were the factors that influenced your decision to participate in clinical trials? |
| 1. **Subject two: Experience of participating in a clinical trial** | |
| 2.1 | What was your experience of being in a clinical trial? |
| 2.2 | What kind of benefits would you like to gain from being in a clinical trial? |
| 2.3 | What are your expectations about clinical trials? |
| 2.4 | What are your concerns about clinical trials? |
| 2.5 | Did you encounter any difficulties during your participation? Do you have any intention of withdrawing from the trial?  (if the answer is “yes”, then ask “Under what circumstances would you withdraw from the trial? Did you eventually withdraw? Why did you stick to it? / What was the reason for your withdrawal?” If the answer is “no”, then ask “What factors do you think are most important in clinical trials?”) |
| 2.6 | Do you think the treatment you received in the trial is helpful for your condition? If so, how is it helpful? Are you satisfied with the experience of participating in the trial? Why? |
| 2.7 | Will you recommend your relatives and friends to participate in clinical trials of acupuncture in the future? What are your expectations or suggestions for the current clinical trials of acupuncture? |
| 1. **Subject three: Experience of previous acupuncture treatment** | |
| 3.1 | Have you experienced any discomfort in the process of acupuncture treatment? If so, what kind of discomfort? Did this discomfort disappoint you at that time? |
| 3.2 | Have you ever discontinued receiving acupuncture during a course of treatment?  (If so, what caused you to discontinue treatment? If not, what beliefs have kept you going?) |
| 3.3 | What factors do you most value when choosing acupuncture to treat your health condition? |
| 3.4 | How do you think the process of doctor-patient communication between acupuncturists and patients affects the improvement of your condition? |
