# Supplementary material for: Factors influencing participant compliance in acupuncture trials: An in-depth interview study
Source: PLoS One. 2020 Apr 16;15(4):e0231780. doi: 10.1371/journal.pone.0231780 (PMC7162473; doi:10.1371/journal.pone.0231780)
Supplement: Attachment — Submitted filename: Response to the author queries_0215.docx [file pone.0231780.s003.docx]

Dear Editor Vaingankar,

Thank you for the opportunity to revise our manuscript; the comments and suggestions are very pertinent and helpful. We have revised the manuscript and responded to the comments point by point as outlined below, and we believe the manuscript is much improved as a result.

Please let us know if there are any further amendments you require for the manuscript.

We look forward to your reply.

Kindly regards,

Huijuan Cao and the author team

**Response to the author queries**

**Reviewer #1:**

1.In the part of study design, 10 samples were described directly. It maybe suitable for the qualitative methods, therefore, it is needed to be revised.
RE: Thank you for pointing out that. We moved the below sentence to the Results section.

*“In total, 10 of the 40 people contacted agreed to take part (25% recruitment rate), and all participants completed the study (0% attrition).”*

2.Only participants who had participated in the study were interviewed, so the attitudes of those who were eligible but refused to participate and those who dropped out were also important factors.
RE: Yes, we agree with you, and have raised this as a limitation of this study, as mentioned in the discussion section. Unfortunately, due to the pragmatics of recruitment to the qualitative study we were not able to contact individuals who had declined participation in the original acupuncture trials. “*A limitation of our study was targeting participants who had previously achieved good compliance in their respective trials, as their opinions may not fully reflect those of participants who declined or withdrew from study participation. However, as the purpose of the current qualitative study was to better understand actual factors that influenced a person’s decision as to why they decided to take part and continue in an acupuncture trial, we would not have captured these viewpoints from those who had declined to participate in the original trials.*” As a follow-on to this, we have suggested in the following section on ‘Implications for future research’ that in future studies “*interviews with both participants who withdrew from clinical trials prior to completion…, to better understand the factors influencing patient compliance from the perspective of all trial stakeholders*” (in Discussion section).

3.The interview outline is not given in this paper, which needs to be supplemented.
RE: Sorry we did not make this clear. The outline of the interview was attached as Appendix 2.

4.To provide a new perspective, the new vision should be more prominent and specific description in the discussion, enhance the innovation of the article.

Thank you for the comments. We have added to the text to further discuss the two main points we raised in this study. Among the four themes we found according to the interview, some both supported and added to previous research findings. One new perspective we found was that the response cost of trial participation may influence the decision to continue with treatment. Aspects of response cost highlighted by the interviewees included time spent attending and receiving treatment, as determined by the frequency and number of sessions. Here we added the below texts: “*According to the results of a UK national survey in 2012 [10], 72.56% of males and 76.99% of females seeking acupuncture treatment are 24-64 years old, with half under 45 years old. Since current acupuncture treatment duration in trials varies from once daily to at least twice weekly excluding weekend days [11], it may cause difficulties for employed participants to attend treatment appointments during their working hours.*” Another point is that some of the concerns and expectations of patients participating in RCTs were related to patients' acceptance of the acupuncture trial. It suggested that we should consider the pain sensation caused by acupuncture operation itself when studying patients' compliance of the acupuncture trial. Here we added that “*Previous studies [12, 13] have already found acupuncture perception had a tendency to be rated higher when needling inserted deep; thus, there may be a relation between the depth of needle insertion and perceived effectiveness of the acupuncture treatment [14, 15]. As such, depth of needling should be considered as a factor in the design of future acupuncture research.*”

**Reviewer #2:**

1.Why only chose the person who had participated the trials before? Why had not chosen the person who rejected to participate the trials? Please justify
RE: Thank you for the comment, which was also raised by Reviewer 1. Please refer to our response to Reviewer 1, comment #2 above. The reason we recruited interviewee from those who participated in previous trial is to better understand the real factors that influence their decisions on.

2. They are difference types of acupuncture trials. How the trials types affected the perceptions of participants?
RE: Sorry we did not clearly clarify that. Our use of “acupuncture clinical trials” in the text referred to randomized controlled trials. We have now amended the text to clarify this, expressing the trial design as randomized controlled trials of acupuncture” in the text.

3. What are the implications for recruitment for acupuncture trials in the future? Please clarify.
RE: Our study found treatment effectiveness, the pragmatics of attending treatment sessions, and the expertise and attitudes of acupuncturists were determining factors of participation and compliance in acupuncture. Thus, the implications for future studies are focused on treatment strategy and doctor-patient communication. For recruitment, we suggested that *future studies should enhance the publicity of trial information*, since *interviewees indicated a lack of prior knowledge of clinical trials*. Also, “*Posters and official Wechat Subscription could be used to publicly and freely disseminate detailed trial information*”. These were all mentioned in the Discussion section. Thank you!

4. In INTRODUCTION, please clarify the current challenges in recruiting trial participant for acupuncture trials
RE: Thank you for the suggestion. We have now added text to describe the challenges for acupuncture trials in the very beginning of the main text (as shown below). More in-depth detail has been described in our previous paper entitled “Attitude and willingness of attendance for participating in or completing acupuncture trials: a cross-sectional study”, which we have cited as the 7th reference of this manuscript.

*“Randomized controlled trials (RCTs) are established as the gold standard to determine intervention efficacy, due to their rigorous design. However, the delivery of an intervention within the structure of an RCT may require participants to follow a sometimes restrictive treatment strategy, which may be contrary to their personal treatment intentions. As a non-drug therapy, the degree of acceptance of acupuncture therapy may be affected by patients' values and preferences. If the patients were randomly assigned to an acupuncture group but did not prefer the treatment regime, this may lead to a higher* *rate of trial withdrawal or intervention attrition.”*

5. In addition, your findings are specific to China?

RE: As is inherent to qualitative research, our findings cannot be generalized outside of our study population. However, we believe our findings provide generic trial information, relevant to researchers regardless of country of origin. We have added the following text to the ‘Limitations; section to explain this: “A further limitation, inherent to qualitative research, is that our findings relating to treatment effectiveness, the pragmatics of attending treatment sessions, and the expertise and attitudes of acupuncturists cannot be generalized outside of our study population. However, while there may be differences between China and other countries regarding patients’ attitudes to these issues, we believe our underlying results highlight generic trial recruitment and compliance issues, and will provide useful information for researchers to improve these study issues, regardless of country of intervention delivery.”

**Additional comments:**

1. Provide further background on compliance to acupuncture trial among Chinese and other races and why this is relevant in medicine

RE: We had previously conducted a literature review, summarising missing data in Chinese acupuncture clinical trials for the period 2006-2015, exploring the influence of missing data on study outcomes through secondary analysis. We have cited cite this paper as the first reference of this manuscript. In response to your comment, we have further added key results of that review in the Background section, to clarify the issue of patient compliance in acupuncture trials.

2. Elaborate further on measures that were taken to reduce selection bias through refusals in this study. Given that this study set out to understand compliance related perspectives of clinical trial patients, by including only those who had good compliance and had repeatedly participated in other trials in the past, has several implications for this study. The issue of non-compliance may not have been adequately explored. Authors should comment on these in the discussion adequately.

RE: Thank you. As per our reply to the previous two reviewers who also raised this point, , we agree that our participant sample may not reflect the attitudes of those who did not have good compliance to acupuncture trials, and have added text to address this limitation, as outlined in our response to Reviewer 1, comment #2 above. We also suggested future studies to involve “*both participants who withdrew from clinical trials prior to completion, and the acupuncturists or researchers directly involved in the trials,* *to better understand the factors influencing patient compliance from the perspective of all trial stakeholders*”.

3. Elaborate on whether saturation was adequately achieved in this study. 30 of the 40 approached had refused participation. Were there other eligible patients that the study team could have approached had saturation not been reached. The 10 participants also seem to be quite heterogenous in their backgrounds. It is also not explained what type of trials they had participated in for example, whether there were of long/ short duration, included invasive procedures or were meant for specific health conditions. All these will have bearings on the qualitative content. Authors should elaborate on these points in the context of data / thematic saturation and implications of the findings.

RE: Sorry we did not make this clear. It was not that 30 of the 40 refused participation, but rather that we only contacted 10 of the 40. As we mentioned in the methods section, “*Participants were continuously sampled from the convenience sample, with sample size determined according to the principle of information saturation. Recruitment was terminated when no new themes were derived from the interview data.*” We contact them one by one, and the recording of the previous interviewee was transcribed and analyzed before we contact to the next person. “*When the analysis of the 9th and 10th interview produced no new codes or concepts, we determined that data saturation had been reached, and no further participants were recruited.*” We have added this sentence to the first paragraph of the Results section.

**Journal Requirements:**

1. Please ensure that your manuscript meets PLOS ONE's style requirements, including those for file naming. The PLOS ONE style templates can be found at
http://www.journals.plos.org/plosone/s/file?id=wjVg/PLOSOne_formatting_sample_main_body.pdf and http://www.journals.plos.org/plosone/s/file?id=ba62/PLOSOne_formatting_sample_title_authors_affiliations.pdf

RE: We have revised the manuscript according to the PLOS ONE style.

2.  Please include captions for your Supporting Information files at the end of your manuscript, and update any in-text citations to match accordingly. Please see our Supporting Information guidelines for more information: <http://journals.plos.org/plosone/s/supporting-information>.

RE: We have included the captions of the Supporting Information files at the end of the manuscript, also we revised the in-text citations as S1 Table and S2 Table.

3. We note that you have indicated that data from this study are available upon request. PLOS only allows data to be available upon request if there are legal or ethical restrictions on sharing data publicly. For information on unacceptable data access restrictions, please see http://journals.plos.org/plosone/s/data-availability#loc-unacceptable-data-access-restrictions.
In your revised cover letter, please address the following prompts:
a) If there are ethical or legal restrictions on sharing a de-identified data set, please explain them in detail (e.g., data contain potentially identifying or sensitive patient information) and who has imposed them (e.g., an ethics committee). Please also provide contact information for a data access committee, ethics committee, or other institutional body to which data requests may be sent.
b) If there are no restrictions, please upload the minimal anonymized data set necessary to replicate your study findings as either Supporting Information files or to a stable, public repository and provide us with the relevant URLs, DOIs, or accession numbers. Please see http://www.bmj.com/content/340/bmj.c181.long for guidelines on how to de-identify and prepare clinical data for publication. For a list of acceptable repositories, please see http://journals.plos.org/plosone/s/data-availability#loc-recommended-repositories.
We will update your Data Availability statement on your behalf to reflect the information you provide.

RE: Data cannot be shared publicly because of recordings of the conversations are private, which contain potentially identifying and personal information. Data are available from the Ethics Committee of Dong Zhi Men Hospital (contact via Dr.Yu) for researchers who meet the criteria for access to confidential data.

The data underlying the results presented in the study are available from Dr. Changhe Yu (yakno2@163.com), via below mail address:

Department of massage, Dong Zhi Men Hospital,

No. 5 of Haiyuncang Road, Dongcheng District,

Beijing, 100701

China

4. Please provide additional details regarding participant consent. In the ethics statement in the Methods and online submission information, please ensure that you have specified (1) whether consent was informed and (2) what type you obtained (for instance, written or verbal). If your study included minors, state whether you obtained consent from parents or guardians. If the need for consent was waived by the ethics committee, please include this information.

RE: We’ve mentioned in the Methods section that “*Potential participants were informed about the researchers’ background, occupation and credentials. They were given an information sheet which described the purpose of the research, and advised by the researcher that they could withdraw from the study at any time, and request their audio recordings to be destroyed. Those in agreement then signed a written consent form prior to their interview taking place*.”

5. Thank you for stating the following in the Acknowledgments Section of your manuscript:

Hui-Juan Cao is supported by the National Natural Science Foundation of China (81804000) and the Beijing Municipal Organization Department (2017000020124G292).

We note that you have provided funding information that is not currently declared in your Funding Statement. However, funding information should not appear in the Acknowledgments section or other areas of your manuscript. We will only publish funding information present in the Funding Statement section of the online submission form.

Please remove any funding-related text from the manuscript and let us know how you would like to update your Funding Statement. Currently, your Funding Statement reads as follows:

The funders had no role in study design, data collection and analysis, decision to publish, or preparation of the manuscript.

RE: Thank you! We removed the funding information from the manuscript. Please update the Funding Statement as “Hui-Juan Cao is supported by the National Natural Science Foundation of China (81804000) and the Beijing Municipal Organization Department (2017000020124G292). Jian-Ping Liu is supported by the Key project of the National Natural Science Foundation of China (No.81830115). The funders had no role in study design, data collection and analysis, decision to publish, or preparation of the manuscript.”
